# Thirty Ways to Improve the Health of the World's Poorest People

**DOI:** 10.1371/journal.pmed.0040310

**Published:** 2007-10-23

**Authors:** The PLoS Medicine Editors

## Abstract

The editors discuss a special collection of articles in*PLoS Medicine* that aims to highlight the profound influence of poverty upon health.

*“The poor fare worse than the better-off almost everywhere and with respect to nearly every indicator” [1]*

If all the different types of health inequalities that have been documented, such as male–female, urban–rural, or the variations in health across ethnic groups, the largest health gap is between the rich and the poor [1]. Today *PLoS Medicine* publishes a special collection of articles that aims to highlight the profound influence of poverty upon health and to debate the most effective mechanisms to redress health disparities between the “haves” and “have-nots.”

Our collection is part of the Council of Science Editors' Global Theme Issue on Poverty and Human Development, involving 231 medical and scientific journals in developed and developing countries, including *PLoS Biology* and *PLoS Neglected Tropical Diseases.* All of the new articles with a link to poverty in these three PLoS journals have been collected together at http://collections.plos.org/poverty.php, along with a selection of previously published articles from all of the PLoS journals.

The Global Theme Issue is being highlighted at a special media event at the United States National Institutes of Health's Fogarty International Center, introduced by National Institutes of Health director Elias Zerhouni. The Council of Science Editors has urged all participating journals to make their poverty theme issues freely available—if these journals comply, the end result should be an unprecedented, publicly accessible collection of materials (available at http://www.councilscienceeditors.org/globalthemeissue.cfm).

The editors of each journal were asked to nominate what they believed to be the most important research article in their theme issue. An expert panel at the Fogarty International Center then chose a handful of the best papers and invited the authors to present their work at the event (a webcast of the event is available at http://videocast.nih.gov/). We are delighted that a *PLoS Medicine* paper by Sheri Weiser and colleagues was chosen, since it is a powerful reminder of how economic deprivation may drive people to take risks with their health [2].

Weiser and colleagues examined the association between food insufficiency (lacking an adequate food supply to meet daily needs) and sexual risk-taking in Swaziland and Botswana. Impoverished women in these countries often lack control over resources, including the household food supply, and are often responsible for taking care of children and household members who are elderly or ill. Engaging in sex may be a way to obtain food for themselves and their families. In their two-country survey, the researchers found that 32% of women and 22% of men experienced food insufficiency in the preceding year, and that food insufficiency was associated with increased HIV risk behaviors, especially in women. Such behaviors included inconsistent condom use, exchanging sex for money, food, or other resources, and intergenerational sex (having had sexual relations with a partner 10 or more years older or younger). “The message of the study is clear,” says Nigel Rollins in his accompanying Perspective [3]. “In the absence of adequate food for oneself or one's family, individuals will forfeit long-term personal safety to survive today.”

This new study is one of several articles in today's special collection in *PLoS Medicine* that shed light on the mechanisms underlying the link between poverty and ill health. For example, in their study of urban low-income housing complexes in Boston, US, Gary Bennett and colleagues found that living in a neighborhood that is perceived to be unsafe at night is a barrier to regular physical activity among low-income urban women [4]. Unsafe housing is also one explanation for the poor health status of people living in urban slums in the developing world, along with overcrowding and inadequate access to safe water, sanitation, and other infrastructure, argue Alon Unger and Lee Riley in their Essay [5]. And Brigit Obrist and colleagues remind us that a crucial reason why the poor fare worse than the rich when it comes to their health is their reduced access to and use of health services [6]. Their conclusion is in line with that of a recent World Bank Report on socioeconomic differences in health in the developing world: “The poor use health services less, have less adequate health-related behaviors, and are disadvantaged with respect to other determinants of health status” [1].

Building a knowledge base on how poverty leads to illness is a crucial first step in determining the policies that could boost the health of the poor. If hunger puts poor women at risk of HIV infection, for example, then program planners surely have a duty, says Rollins, to “consider hunger alleviation as a central component of HIV prevention programmes” [3].

In addition to hunger alleviation, what are the other interventions that could transform the health of the world's poorest people? We put this question to a wide range of commentators worldwide—from eminent global health advocates, such as Jeffrey Sachs and Paul Farmer, to health reporters, activists, health researchers, and those living in poverty themselves. Specifically, we asked them to answer, in around 50 words, the question, “Which single intervention would do the most to improve the lives of those living on less than $1 a day?” The 30 responses are published in our *PLoS Medicine* Debate [7].

The responses are striking in three ways. First, almost all of the suggested interventions are low cost and low tech. The global community easily has the financial and technical means to scale up all of these interventions immediately—it has more than enough resources, for example, to distribute insecticide-treated bed nets and artemisinin-based combination therapy for malaria, train community health workers, promote breast-feeding, and vaccinate all children.

Second, the responses from members of poor rural communities in Ayacucho, Peru, remind us that the views of the poor themselves about how to improve their health may diverge from the views of international development experts. Community members talked about the importance of housing, food, family, and social interactions—a view of health promotion that goes far beyond a strictly biomedical approach.

Finally, many of the responses highlight the importance of the rich world fulfilling its obligations to the global poor through, for example, cash transfers from high- to low-income countries or committing 1% of gross domestic product to international development assistance. Indeed, one message that arises from the Council of Science Editors' Global Theme Issue is that our global interconnectedness is surely at the heart of addressing inequities in health that have an economic basis. “Only when (and if) the “haves” develop genuine empathy for the “have-nots,” says Solomon Benatar, Professor of Medicine at the University of Cape Town, South Africa and a member of the editorial board of *PLoS Medicine*, “and come to acknowledge their own long-term interdependence with all other humans, will the global economy be improved to any significant advantage for the desperately poor” [7].
